# Variability in lutetium-177 SPECT quantification between different state-of-the-art SPECT/CT systems

**DOI:** 10.1186/s40658-020-0278-3

**Published:** 2020-02-11

**Authors:** Steffie M. B. Peters, Sebastiaan L. Meyer Viol, Niels R. van der Werf, Nick de Jong, Floris H. P. van Velden, Antoi Meeuwis, Mark W. Konijnenberg, Martin Gotthardt, Hugo W. A. M. de Jong, Marcel Segbers

**Affiliations:** 1 Department of Radiology and Nuclear Medicine, Department of Radiology and Nuclear Medicine, Radboud University Medical Center, P.O. Box 9101, 6500 HB Nijmegen, The Netherlands; 2 Department of Radiology and Nuclear Medicine, University Medical Center Utrecht, Utrecht, The Netherlands; 3 Department of Radiology and Nuclear Medicine, Erasmus MC, Rotterdam, The Netherlands; 4 Department of Radiology, Section of Medical Technology, Leiden University Medical Center, Leiden, The Netherlands

## Abstract

**Background:**

Quantitative SPECT imaging in targeted radionuclide therapy with lutetium-177 holds great potential for individualized treatment based on dose assessment. The establishment of dose-effect relations requires a standardized method for SPECT quantification. The purpose of this multi-center study is to evaluate quantitative accuracy and inter-system variations of different SPECT/CT systems with corresponding commercially available quantitative reconstruction algorithms. This is an important step towards a vendor-independent standard for quantitative lutetium-177 SPECT.

**Methods:**

Four state-of-the-art SPECT/CT systems were included: Discovery™ NM/CT 670Pro (GE Healthcare), Symbia Intevo™, and two Symbia™ T16 (Siemens Healthineers). Quantitative accuracy and inter-system variations were evaluated by repeatedly scanning a cylindrical phantom with 6 spherical inserts (0.5 – 113 ml). A sphere-to-background activity concentration ratio of 10:1 was used. Acquisition settings were standardized: medium energy collimator, body contour trajectory, photon energy window of 208 keV (± 10%), adjacent 20% lower scatter window, 2 × 64 projections, 128 × 128 matrix size, and 40 s projection time. Reconstructions were performed using GE Evolution with Q.Metrix™, Siemens xSPECT Quant™, Siemens Broad Quantification™ or Siemens Flash3D™ algorithms using vendor recommended settings. In addition, projection data were reconstructed using Hermes SUV SPECT™ with standardized reconstruction settings to obtain a vendor-neutral quantitative reconstruction for all systems. Volumes of interest (VOI) for the spheres were obtained by applying a 50% threshold of the sphere maximum voxel value corrected for background activity. For each sphere, the mean and maximum recovery coefficient (RC_mean_ and RC_max_) of three repeated measurements was calculated, defined as the imaged activity concentration divided by the actual activity concentration. Inter-system variations were defined as the range of RC over all systems.

**Results:**

RC decreased with decreasing sphere volume. Inter-system variations with vendor-specific reconstructions were between 0.06 and 0.41 for RC_mean_ depending on sphere size (maximum 118% quantification difference), and improved to 0.02–0.19 with vendor-neutral reconstructions (maximum 38% quantification difference).

**Conclusion:**

This study shows that eliminating sources of possible variation drastically reduces inter-system variation in quantification. This means that absolute SPECT quantification for ^177^Lu is feasible in a multi-center and multi-vendor setting; however, close agreement between vendors and sites is key for multi-center dosimetry and quantitative biomarker studies.

## Introduction

Quantitative SPECT imaging in targeted radionuclide therapy with lutetium-177 (^177^Lu) holds great potential for dosimetry-based individualized treatment and may improve prediction of therapy response, prevention of toxicity effects and treatment follow-up. With the advent of ^177^Lu-PSMA therapy [1–4], it is expected that dosimetry will play a pivotal role in the reliable determination of dose-response relationships in tumors. But also our understanding of biomarker studies and already well-established radionuclide therapies in neuroendocrine tumors [5–9] may profit from optimized quantitative SPECT imaging for sophisticated dosimetry.

SPECT quantification is considered less straightforward than PET quantification [10, 11]. This can be explained by several factors including lower sensitivity due to the necessary use of a collimator, the need for more complicated scatter and attenuation correction [11] and a lower resolution creating partial volume effects. Several studies investigated the quantitative performance of SPECT for a variety of radionuclides, including technetium-99 m (^99m^Tc) [12, 13], indium-111 (^111^In) [14–16], iodine-131 (^131^I) [17], yttrium-90 (^90^Y), or a combination of these [18, 19] and concluded that quantification is possible, be it with certain limitations, for example, with regard to small structures as a result to the partial volume effect. Beauregard et al. looked into the quantitative accuracy of ^177^Lu on one SPECT/CT system [20] and found that this could yield more accurate dosimetry estimates than planar imaging. Hippeläinen et al. compared the results of different ordered subset expectation maximization (OSEM) reconstruction algorithms [21] and concluded that alignment was best when the images were corrected for attenuation, scatter, and detector and collimator response. Various SPECT/CT vendors have responded to the increasing need for SPECT quantification and now commercially offer software packages for quantification of several radionuclides including ^177^Lu [22–24].

However, standardization of protocols such that quantitative results can be reliably compared between systems requires more insight in their quantitative accuracy and performance. This is key for, e.g., multi-center research trials involving absolute SPECT quantification, especially those aimed towards dosimetry. Our previous study compared quantification for SPECT/CT systems from different vendors at different imaging centers for technetium-99 m and showed that standardizing reconstruction decreased inter-system variability [25]. The aim of this study is to extend these findings to ^177^Lu. The quantitative accuracy and inter-system variability of recovery coefficients (RC) were determined using phantom experiments and the effects of lesion volume and reconstruction algorithm on RC were investigated. The results of these comparisons can be used as input for a vendor-independent standard for absolute quantitative SPECT of ^177^Lu.

## Methods

### SPECT/CT systems

Four SPECT/CT systems from two manufacturers were included in this study: a Discovery NM/CT 670 Pro (GE Healthcare, Milwaukee, USA), a Symbia Intevo Bold, and two Symbia T16’s (Siemens Healthineers, Erlangen, Germany) (Table 1). Three out of four systems had commercial software packages for quantification installed, as listed in Table 2.

**Table 1:** Main properties of the dual headed SPECT/CT systems used in this study

System	General Electric Discovery NM/CT 670 Pro	Siemens Symbia Intevo Bold	Siemens Symbia T16 system 1	Siemens Symbia T16 system 2
Imaging center	Leiden University Medical Center	University Medical Center Utrecht	Radboud University Medical Center	Erasmus University Medical Center
SPECT detector	3/8″ NaI crystal 59 PMT* 40 × 54 cm FOV*	3/8″ NaI crystal 59 PMT* 38.7 × 53.3 cm FOV*	3/8″ NaI crystal 59 PMT* 38.7 × 53.3 cm FOV*	3/8″ NaI crystal 59 PMT* 38.7 × 53.3 cm FOV*
CT	16-slice	16-slice	16-slice	16-slice

*PMT photomultiplier tube, *FOV* field of view

**Table 2:** Reconstruction/quantification parameters and processing software used in this study. Quantification packages Q. Metrix, xSPECT Quant, and Broad quantification enable quantitative reconstructions in the scanner software

System	Discovery NM/CT 670 Pro	Symbia Intevo Bold	Symbia T16 system 1	Symbia T16 system 2	All (standardized)
Imaging center	Leiden University Medical Center	University Medical Center Utrecht	Radboud University Medical Center	Erasmus University Medical Center	All
Reconstruction	OSEM^a^ + Evolution with PSF^a^ correction	WCG^a^ + xSPECT with PSF^a^ correction	OSEM^a^ + Flash 3D with PSF^a^ correction	OSEM^a^ + Hybrid Recon V3.0.0 with PSF^a^ correction	OSEM^a^ + Hybrid Recon V3.0.0 with PSF^a^ correction
Quantification	Q.Metrix	xSPECT Quant/Broad Quantification^b^	Manual analysis	Hermes SUV SPECT	Hermes SUV SPECT
Iterations	9^d^	6	4	5	5
Subsets	10	8	8	16	16
Post-reconstruction filter	None	5 mm (Gaussian)	4 mm (Gaussian)	5 mm (Gaussian)	5 mm (Gaussian)
Attenuation correction	CT based^c^	CT based^c^	CT based^c^	CT based^c^	CT based^c^
Scatter Correction	DEW^a^ (170 ± 10%)	DEW^a^ (170 ± 10%)	DEW^a^ (170 ± 10%)	Monte Carlo-based	Monte Carlo-based
Reconstruction voxel size	2.0 × 2.0 × 2.0 mm^3 e^	4.9 × 4.9 × 4.9 mm^3^	4.8 × 4.8 × 4.8 mm^3^	4.8 × 4.8 × 4.8 mm^3^	4.8 × 4.8 × 4.8 mm^3^

^a^*OSEM* ordered subset expectation maximization; *WCG* weighted conjugate gradient, *PSF* point spread function, *DEW* double energy window

^b^ For xSPECT Quant and Broad quantification separate measurements were performed

^c^ Bilinear conversion of HU into attenuation coefficients at 208 keV

^d^ Settings were not according to vendor recommendation but to the literature [13]

^e^ Interpolation to 2.0 × 2.0 × 2.0 mm^3^ voxels by Q. Metrix for quantification, as recommended by the vendor

### Phantom

**Fig. 1:**
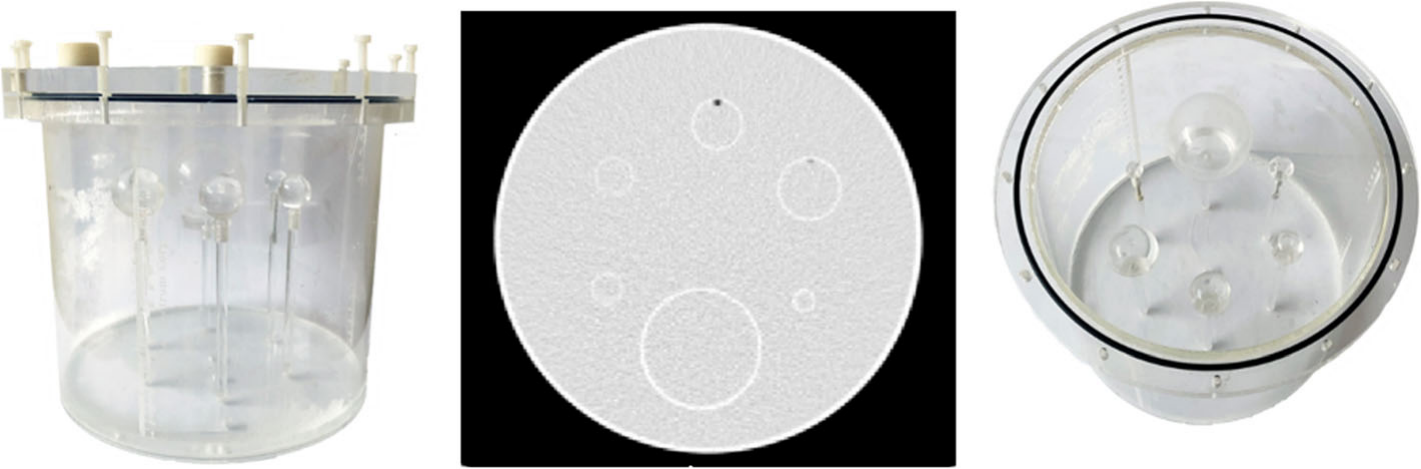
The phantom used to determine the recovery coefficients. The second image is a CT slice of the phantom on which the order of the spheres can be seen

A modified cylindrical Jaszczak phantom (Fig. 1) was used, with a background compartment volume of approximately 6.7 l and regular inserts replaced by 6 spherical inserts representing various lesion sizes with inner diameters (and brief volumes) of 9.9 mm (0.5 ml), 15.4 mm (2.0 ml), 19.8 mm (4.0 ml), 24.8 mm (8.0 ml), 31.3 mm (16.0 ml), and 60.0 mm (113 ml).

The spheres and background compartment were filled with a homogeneous solution of ^177^Lu in water with an activity concentration of approximately 750 kBq/ml for the spheres and 75 kBq/ml for the background compartment, resulting in a sphere-to-background ratio of 10:1. The concentrations were based upon the expected lesion uptake in lutetium therapy [2, 26]. The solution was saturated with a 50 mM ethylenediaminetetraacetic acid (EDTA) solution to prevent precipitation of lutetium. Enabled by the long half-life time of ^177^Lu (*T*_1/2_ = 665 days), user preparation differences were excluded by the study set-up, as the phantom was filled once before being sent around to the participating centers. The time between first and last measurement was 74.7 h, and during acquisition, the measurement time per angle was adjusted for activity decay to obtain similar count statistics for each measurement.

To check for possible quantification differences caused by the use of different dose calibrators, a syringe filled with the same solution with an activity of 29.0 MBq ^177^Lu was measured in each center and compared to the activity measured in the reference center.

### Acquisition

Images were acquired with a Medium Energy General Purpose (MEGP) or Medium Energy Low Penetration (MELP) collimator (Additional file 1: Table S1). Acquisition settings were harmonized across all systems according to MIRD Pamphlet No. 26 [27]: body contour trajectory, a photon energy window of 208 keV (± 10%), adjacent 20% lower scatter window, 2 × 64 projections, a 128 × 128 matrix size, and a reference projection time of 40 s. On each system, the measurement was repeated three times to assess repeatability.

### Reconstruction

Reconstructions were performed with vendor/center specific 3D iterative reconstruction algorithms and quantification packages (Table 2). The reconstructions were performed with scatter correction, CT-based attenuation correction (Additional file 1: Table S2) and resolution recovery, using vendor recommended settings (Table 2). In addition to vendor/center specific reconstructions, all raw data were reconstructed with a vendor neutral-reconstruction algorithm (Hybrid Recon v3.0.0, Hermes SUV SPECT™, Stockholm, Sweden), with standardized reconstruction settings (Table 2) to obtain vendor-neutral quantitative reconstructions for all systems.

### Calibration factor

All SPECT/CT systems were cross-calibrated for ^177^Lu with the in-house dose calibrator according to the manufacturer’s guidelines or to the center’s standard practice (Additional file 1: Table S3). An exception was the Symbia Intevo Bold (xSPECT) quantification calibration, which is different from the other calibration methods, as it makes use of a (by manufacturer) included ^75^Se calibrated sensitivity precision source instead of a ^177^Lu source. All dose calibrators used in this study to cross-calibrate the SPECT systems undergo regular quality control according to national guidelines [28].

To determine the calibration factor for the vendor-neutral quantification method each site performed a calibration according to the guidelines of this particular software vendor. Each site scanned a homogeneous cylindrical phantom with a 6 to 7 l volume and approximately 500 MBq ^177^Lu with the same acquisition protocol as used in the experiments. Volumes of interest (VOIs) were drawn to obtain a calibration factor (CF):

1 $$ \mathrm{CF}\ \left[\frac{\frac{\mathrm{cps}}{\mathrm{ml}}}{\frac{\mathrm{kBq}}{\mathrm{ml}}}\right]=\frac{\left(\frac{\mu\ }{t\bullet n\bullet \nu}\right)}{C}, $$

where *μ* is the mean voxel value in counts in the reconstructed image, *t* is the time per projection, *n* is the number of projections, *ν* is the voxel size and C is the actual activity concentration in the phantom.

### Image analysis

Image analyses were performed using in-house developed software in Python. This script uses the SimpleITK toolkit region growing algorithm to determine the sphere VOI [29, 30]. The VOIs were obtained by applying a 50% threshold of the sphere maximum voxel value with a correction for the background activity [31]:

2 $$ {\mathrm{VOI}}_{\mathrm{thresh},j}=0.5\bullet \left({\mathrm{VOI}}_{\max, j}+{\mathrm{VOI}}_{\mathrm{mean}, bg}\right) $$

where VOI_thresh,*j*_ is the VOI threshold voxel value of sphere *j*, VOI_max,*j*_ is the maximum voxel value in the sphere VOI and VOI_mean,bg_ is the mean voxel value in the background VOI. The background was determined by placing a single cylindrical VOI (diameter 9 cm, 5 cm height) in a uniform region within the phantom. For each sphere VOI, the mean and maximum recovery coefficient (respectively RC_mean_ and RC_max_) were calculated, defined as the mean/maximum imaged activity concentration (*A*_*i*_) over the three consecutive measurements, divided by the actual activity concentration (*A*_*a*_):

3 $$ {\mathrm{RC}}_{\mathrm{mean},j}=\frac{A_{i,j}}{A_{a,j}} $$

4 $$ {\mathrm{RC}}_{\max, j}=\frac{A_{i,\max, j}}{A_{a,j}}\kern0.5em $$

The inter-system variability was assessed for each sphere diameter by the range of the RC over all systems according to:

5 $$ {\mathrm{Range}}_j={\mathrm{RC}}_{j,\max }-{\mathrm{RC}}_{j,\min } $$

where *j* is the sphere diameter. This range was calculated for both the RC_mean_ and RC_max_.

This study included three systems of one vendor (Siemens), all consisting of (almost) equal hardware. Therefore, it was possible to compare quantification differences within one vendor as well, thereby focusing on differences between systems without the influence of their hardware.

### Error analysis

Uncertainties were determined for both the CF and de RC according to the EANM guidelines by Gear et al. [32]. The recovery coefficient curve as a function of sphere volume was fitted with a 3-parameter logistic function.

## Results

### Calibration

Differences in activity in the syringe as determined by the dose calibrator in each center were 1–4% (Table 3).

**Table 3:** Measured differences in activity of a syringe filled with 29.0 MBq ^177^Lu resulting from the use of different dose calibrators as used for the given systems

System	Deviation (compared to reference)
Symbia T16 system 1 (reference)	1.00
Discovery NM/CT 670 Pro	0.96
Symbia Intevo Bold, xSPECT Quant	1.01
Symbia T16 system 2	0.99

Table 4 shows the calibration factors of each system. The error in CF is assumed to be within 5% since it is dominated by the uncertainty in the activity used in the cylindrical phantom. The activity was measured in a dose calibrator with an uncertainty smaller than 5% (Table 3). In addition, the standard deviation in repeated measurements of a 389 voxel-sized VOI used for the average voxel counts μ (Eq. 1) was 0.7%.

**Table 4:** Calibration factor (CF) for each system for vendor-neutral quantification

System	CF for Hermes SUV SPECT [cps/MBq]
Discovery NM/CT 670 Pro	6.2
Symbia Intevo Bold, xSPECT Quant	10.2
Symbia Intevo Bold, Broad Quantification	10.2
Symbia T16 system 1	10.3
Symbia T16 system 2	10.1

### Recovery coefficient

The actual sphere-to-background activity concentration ratio based on dose calibrator measurements was 9.4:1. The median recovery coefficient of the background compartment for the five different vendor-specific reconstructions was 0.97 (range 0.92–1.06).

**Fig. 2:**
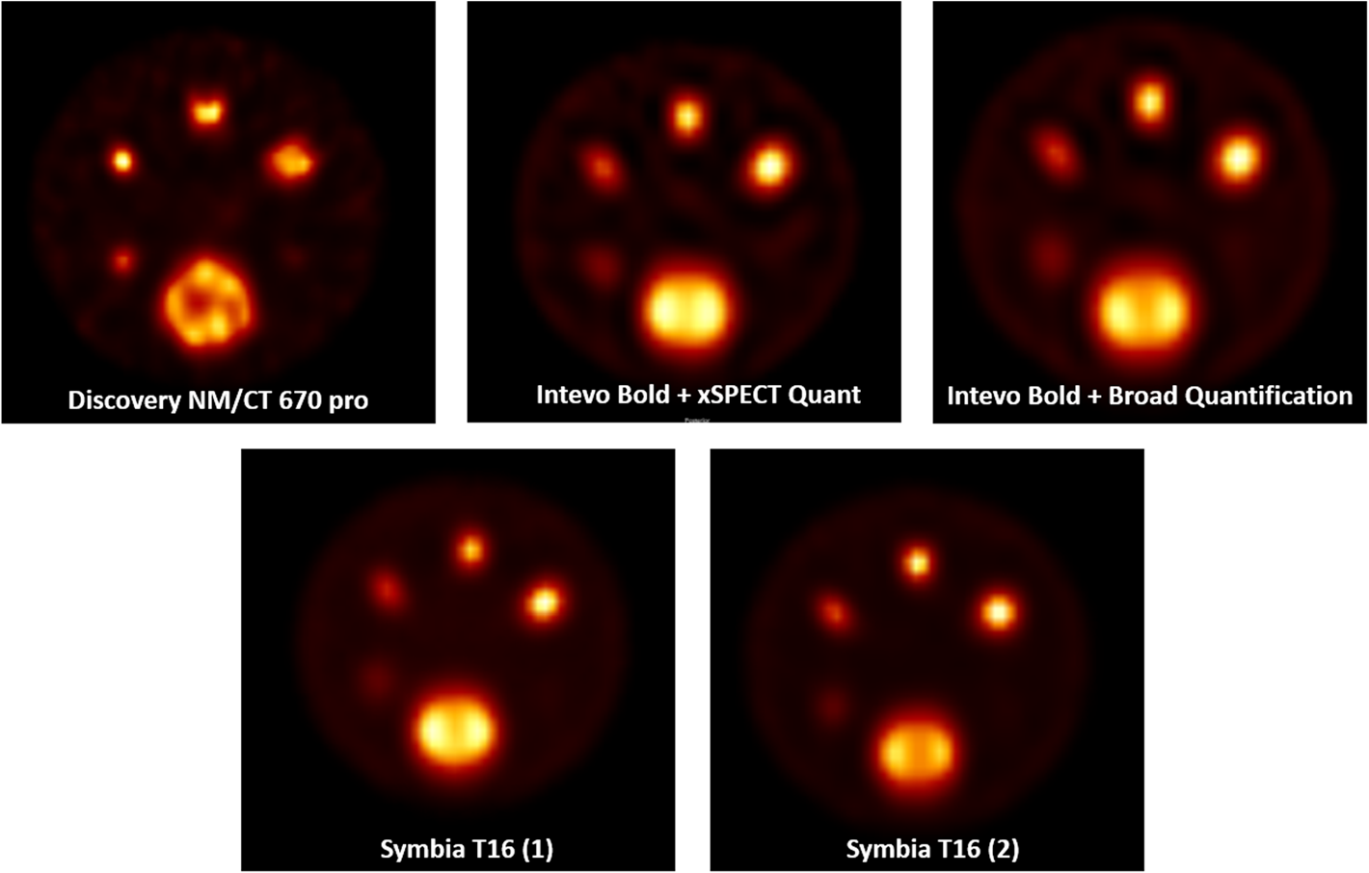
SPECT images of the cylindrical phantom for all systems, reconstructed with vendor-specific reconstruction algorithms

**Fig. 3:**
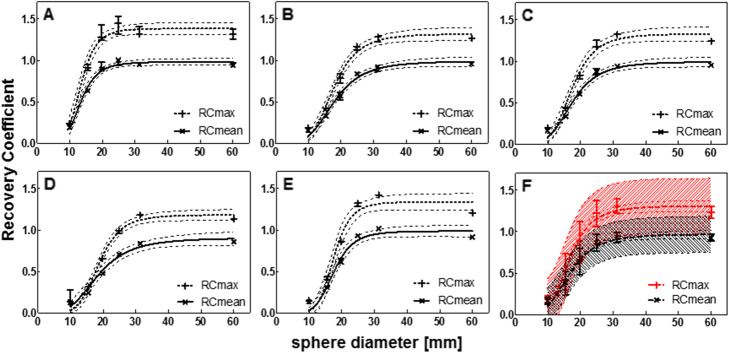
Recovery coefficient as a function of sphere diameter for all systems separately (**a**–**e**) and for all systems combined (**f**), for data reconstructed with a vendor-specific algorithm. Median and range of three repetitive measurements per system. **a** Discovery NM/CT 670 Pro. **b** Symbia Intevo Bold with xSPECT Quant. **c** Symbia Intevo Bold with Broad Quantification. **d** Symbia T16 system 1. **e** Symbia T16 system 2. **f** Mean and standard deviation. All data were fitted with a 3-parameter logistic function (dashed line: 95% CI), for the combined data (**f**) also the 95% prediction interval is indicated (dashed area)

The center-specific SPECT reconstructions are shown in Fig. 2. Due to the low contrast compared to the background, the smallest sphere (9.9 mm diameter) is not or barely visible. The recovery coefficients of the spheres (Fig. 3a–e) decreased with decreasing sphere diameter on all systems. The variability between systems for RC_mean_ is visualized in Fig. 3f by plotting median and range for all systems. A large variability was found for spheres with a diameter ≤ 24.8 mm with a total RC range of up to 0.41 for (resulting in 118% quantification differences between systems) and 0.62 for RC_max_ (139% quantification differences), especially when compared to the largest sphere diameter (60 mm) that showed variability of 0.10 (11%) and 0.19 (15%) for RC_mean_ and RC_max_ respectively.

The RC values were fitted with a 3-parameter logistic function as suggested in the EANM guidelines [32]. The additional parameter was introduced to allow the logistic function to reach asymptotic values different from unity. All curves showed a good correlation with the data (Pearson *R*^2^ > 0.96).

### Effect of reconstruction algorithm on recovery coefficients

**Fig. 4:**
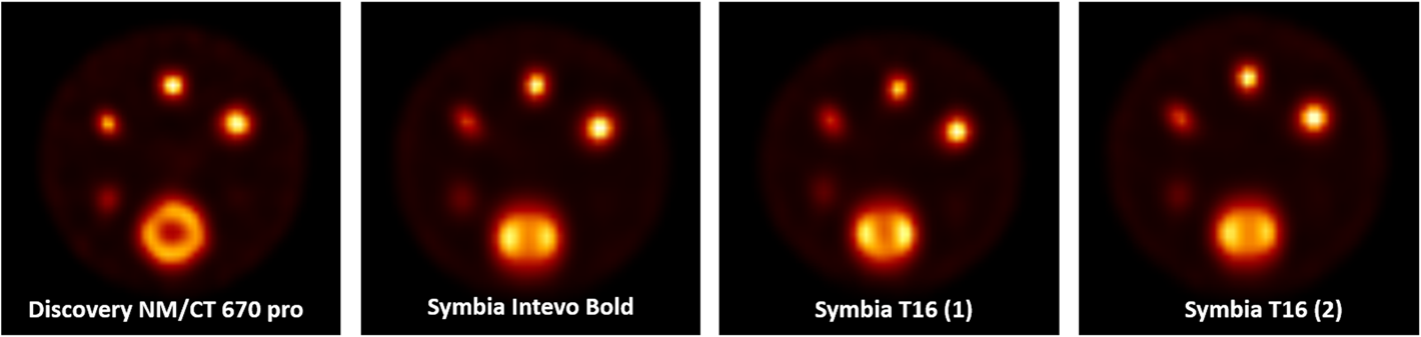
SPECT images of the cylindrical phantom for all systems, reconstructed with a vendor-neutral algorithm

The vendor-neutral SPECT reconstructions are shown in Fig. 4. Using this reconstruction algorithm on all data leads to visually more similar reconstructions. The median recovery coefficient of the background compartment for the four different vendor-specific reconstructions was 1.03 (range 0.91–1.07).

**Fig. 5:**
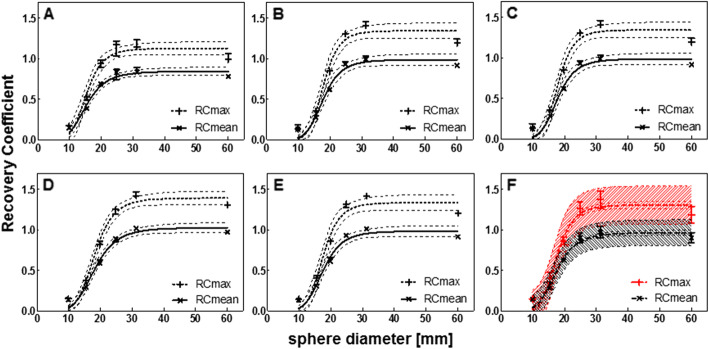
Recovery coefficient as a function of sphere diameter for all systems separately (**a**–**e**) and for all systems combined (**f**), for data reconstructed with a vendor-neutral algorithm. Median and range of three repetitive measurements per system. **a** Discovery NM/CT 670 Pro. **b** Symbia Intevo Bold with xSPECT Quant. **c** Symbia Intevo Bold with Broad Quantification. **d** Symbia T16 system 1. **e** Symbia T16 system 2. **f** Mean and standard deviation for all systems combined. All data were fitted with a 3-parameter logistic function (dashed line 95% CI), for the combined data (**f**) also the 95% prediction interval is indicated (dashed area)

**Fig. 6:**
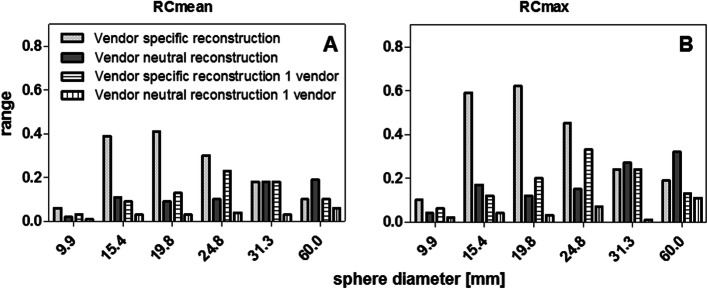
Comparison in range over all systems in RC_mean_ (**a**) and RC_max_ (**b**) per sphere diameter for data reconstructed with a vendor-specific algorithm versus a vendor-neutral algorithm. Third and fourth columns give the same information but for systems of only one vendor, thus consisting of equal system hardware

Figure 5 shows the recovery coefficient per system for the vendor-neutral reconstructions. By comparing Fig. 3 (vendor-specific) to Fig. 5 (vendor-neutral), a decrease in inter-system variability can be seen. This was confirmed by a large decrease in range for all sphere diameters ≤ 24.8 mm for both RC_mean_ (0.9 to 0.11, resulting in quantification differences between systems of up to 38%) and RC_max_ (0.12 to 0.17, resulting in quantification differences of up to 46%) (Fig. 5). Figure 6 shows the inter-system variability (RC range) for vendor-neutral and vendor-specific reconstructions. For the two largest spheres, the inter-system variability slightly increased compared to the vendor-specific reconstruction, resulting in quantification differences of up to 21%. When comparing systems within the same vendor, the inter-system variations result in quantification differences for all sphere sizes of up to 11% for RC_mean,_ and 12% for RC_max_. This illustrates the large effect of the system hardware on quantification differences.

## Discussion

This study shows that standardizing reconstruction settings decreases inter-system variability for quantification of ^177^Lu. This has important implications for multi-center studies where quantification plays an important role in dosimetry studies.

In order to eliminate preparation differences, the phantom was prepared only once and sent around to all participating centers. Furthermore, differences in activity measurements were monitored by measuring a syringe filled with a known amount of ^177^Lu at each center and using the first center as a reference. Since one of the purposes of our study was to illustrate the differences in quantification between centers, the measured activity differences were not corrected to determine the RC. However, it was found that for one center, the deviation in activity was 4%, which of course could also affect the final quantification on the SPECT/CT system since the cross-calibration for ^177^Lu used for quantification was based on measurements on the center’s own dose calibrator.

An adaptation of the Jaszczak phantom was used to determine the RC for different sphere diameters. The varying sphere diameters represent different lesion sizes and can give an estimate of the expected RC values for these lesions. For quantification as input for dosimetry, one could consider using a correction factor for smaller lesions, which show RC values well below a value of 1.0 [32].

In this study, reconstruction settings were used that are applied in clinical practice for each center. These reconstruction settings were based mainly on the recommendations of the vendor, with possible adjustments by the center itself. These settings might not be ideal for ^177^Lu quantification, and the number of iterations used in reconstruction, as well as the possible additional use of a post-reconstruction filter, might influence the quantification [33, 34]. Furthermore, Dewaraja and colleagues [35] pointed out that post-reconstruction filtering is not desirable for quantifying total target activity, but acceptable when calculating 3D doses such as dose-volume histograms. This indicates that the reconstruction and post-reconstruction settings used in this study could be optimized further for quantification, possibly leading to better alignment in quantification between different centers and systems.

Due to the low contrast compared to the background, the smallest sphere (9.9 mm diameter) was barely visible. This limitation in system spatial resolution is in line with the difficulty in quantification found by other studies [18, 19]. Therefore, the recovery coefficients for this sphere volume should be interpreted with caution. Although it was shown that the range in RC between systems decreased from the second-smallest to the smallest sphere, it is expected that this is mainly due to the fact that the used segmentation method, a threshold based on 50% of the maximum voxel value, was not able to delineate a reliable VOI for the smallest sphere.

Image analysis was performed using an automated Python script that uses a background-corrected 50% isocontour method based on the study of Frings and colleagues. Although a 42% isocontour method shows good recovery for both PET [36] and SPECT [13], the background-corrected 50% isocontour method was chosen because of its high repeatability for PET in a multicenter settings [37] and to align our results as much as possible with the already existing standards for PET quantification [38]. However, a recently published study by Ryu et al. [39] showed that the line profiles over active spheres of reconstructed SPECT images (using ^99m^Tc and ^177^Lu) showed a very different profile than the same spheres measured on PET (using ^18^F and ^68^Ga). This indicates that a 50% isocontour method might not be the most ideal solution threshold for contouring in SPECT and that a lower threshold might be more appropriate as demonstrated by Collarino et al. [13]. However, the goal of this study was to assess the effects of different quantitative SPECT imaging systems, independent of the applied delineation method.

On the largest sphere, a low recovery coefficient was found, especially for reconstruction with the vendor-neutral algorithm (Hermes SUV SPECT). This can most probably be explained by the use of a 50% threshold for the VOI delineation, and a contribution of the Gibbs artifact, which is clearly visible in Figs. 2 and 4. A typical strategy in handling Gibbs ringing artifacts is to reduce them with compromised resolution [40, 41]. This reduction can be achieved by blurring the input image so that the data do not contain high-frequency components, by reconstruction other than PSF or by using post-reconstruction Gaussian filtering. Although not the goal of this study, reducing the Gibbs artifact might contribute to better alignment in quantification between centers. Additionally, for the Discovery NM/CT 670 Pro and the Symbia T16 system 1, the low RC for the largest sphere might be explained by the number of iterations and subsets in the reconstruction settings. This was according to the center’s standard settings which are based on vendor recommendations and other literature on reconstruction settings [13]. However, increasing the number of iterations might increase recovery and thereby improve quantification accuracy.

The values for RC_max_ are systematically over 1 for spheres with a diameter > 24.8 mm. Although an overshoot for RC_max_ was also found in other studies [13, 42], it was even higher in this study (1.3 ± 0.2). This overshoot is not a statistical error but is most probably the result of the resolution recovery algorithm that was used during reconstruction. This algorithm was used in the standard reconstruction as recommended by the vendor.

For spheres with a diameter < 25 mm, RC quickly decreases as expected, mainly as a result of partial volume effects. For GE Evolution reconstruction, no post-reconstruction Gaussian smoothing filter was applied, which might explain a higher RC for small sphere diameters. This high RC is also reflected in the large inter-system variations for small sphere diameters.

In this study, both RC_mean_ and RC_max_ were determined and compared between systems. The results could be used to work towards a normalization between centers and systems. Depending on the application, the choice for either RC_mean_ or RC_max_ as a tool for standardization could be more applicable. For example in ^18^F-FDG PET quantification, evaluation of treatment response is of main interest. In this case, this could be evaluated by using the SUV_max_ and therefore standardization based on RC_max_ would be a logical choice. For quantification of ^177^Lu, however, the most obvious application would be for the use in dosimetry for radionuclide therapies with ligands such as ^177^Lu-PSMA for prostate cancer or ^177^Lu-DOTATATE/DOTATOC for neuroendocrine tumors. This means a VOI is needed to determine the accumulated activity, in which the mean voxel value is the most relevant parameter. Therefore, we suggest that for normalization of ^177^Lu quantification, the RC_mean_ could be used as a tool for standardization between centers.

Although this study provides valuable insight in quantification differences between systems, it only compared four SPECT/CT systems (five quantification methods). For one vendor, three systems and four quantification methods were included in this study. We showed that by eliminating the effect of system hardware, the inter-system variability was greatly reduced. Standardizing the reconstruction algorithm led to a further decrease in intersystem variability. It is therefore paramount to harmonize SPECT/CT image reconstructions in a multi-center/multi-vendor setting. These data can be used as input to work towards a standard for quantification of ^177^Lu, but it needs to be expanded to more centers and/or systems, preferably also across borders. We suggest that further standardization could improve the alignment of quantification between different SPECT/CT systems, comparable to the EARL accreditation program for ^18^F-FDG PET/CT [42]. Still, it is important to realize that variability in quantification between SPECT/CT systems will probably be larger than those found in this study, due to, for example, patient positioning and patient size.

## Conclusion

This study shows that absolute SPECT quantification for ^177^Lu is feasible in a multi-center and multi-vendor setting. With standardized acquisition protocols but center-specific data reconstruction algorithms, the inter-system variability (range in RC between systems) was as large as 0.41 and 0.62 for RC_mean_ and RC_max_, respectively. Standardizing reconstruction decreased this range to 0.19 and 0.32, respectively. Close agreement between vendors and sites is key for multi-center dosimetry and quantitative biomarker studies. This study serves as an important step towards a vendor-independent standard for absolute quantification in SPECT/CT of ^177^Lu.

## Supplementary information

**Additional file 1:Table S1.** Characteristics of ME collimators for all used SPECT/CT systems. **Table S2.** Acquisition settings of low dose CT protocols used for attenuation correction. **Table S3.** Cross-calibration protocols for dose calibrators to SPECT/CT system.

## Data Availability

The datasets used and/or analyzed during the current study are available from the corresponding author on reasonable request.
