# Correction to: Variability in lutetium-177 SPECT quantification between different state-of-the-art SPECT/CT systems

**DOI:** 10.1186/s40658-021-00399-y

**Published:** 2021-08-18

**Authors:** Steffie M. B. Peters, Sebastiaan L. Meyer Viol, Niels R. van der Werf, Nick de Jong, Floris H. P. van Velden, Antoi Meeuwis, Mark W. Konijnenberg, Martin Gotthardt, Hugo W. A. M. de Jong, Marcel Segbers

**Affiliations:** 1 Department of Radiology and Nuclear Medicine, Department of Radiology and Nuclear Medicine, Radboud University Medical Center, P.O. Box 9101, 6500 HB Nijmegen, The Netherlands; 2 Department of Radiology and Nuclear Medicine, University Medical Center Utrecht, Utrecht, The Netherlands; 3 Department of Radiology and Nuclear Medicine, Erasmus MC, Rotterdam, The Netherlands; 4 Department of Radiology, Section of Medical Technology, Leiden University Medical Center, Leiden, The Netherlands

**Correction to: EJNMMI Phys 7, 9 (2020)**

**https://doi.org/10.1186/s40658-020-0278-3**

Following publication of the original article [1], it was reported that the sphere volumes defined in the original article should be adjusted. The correct inner diameters (and volumes) of the spherical inserts were: 9.9 mm (0.5 ml), 15.4 mm (2.0 ml), 19.8 mm (4.0 ml), 24.8 mm (8.0 ml), 31.3 mm (16.0 ml) and 60 mm (113 ml). Figures 3, 5 and 6 have been adjusted accordingly.

**Fig. 3:**
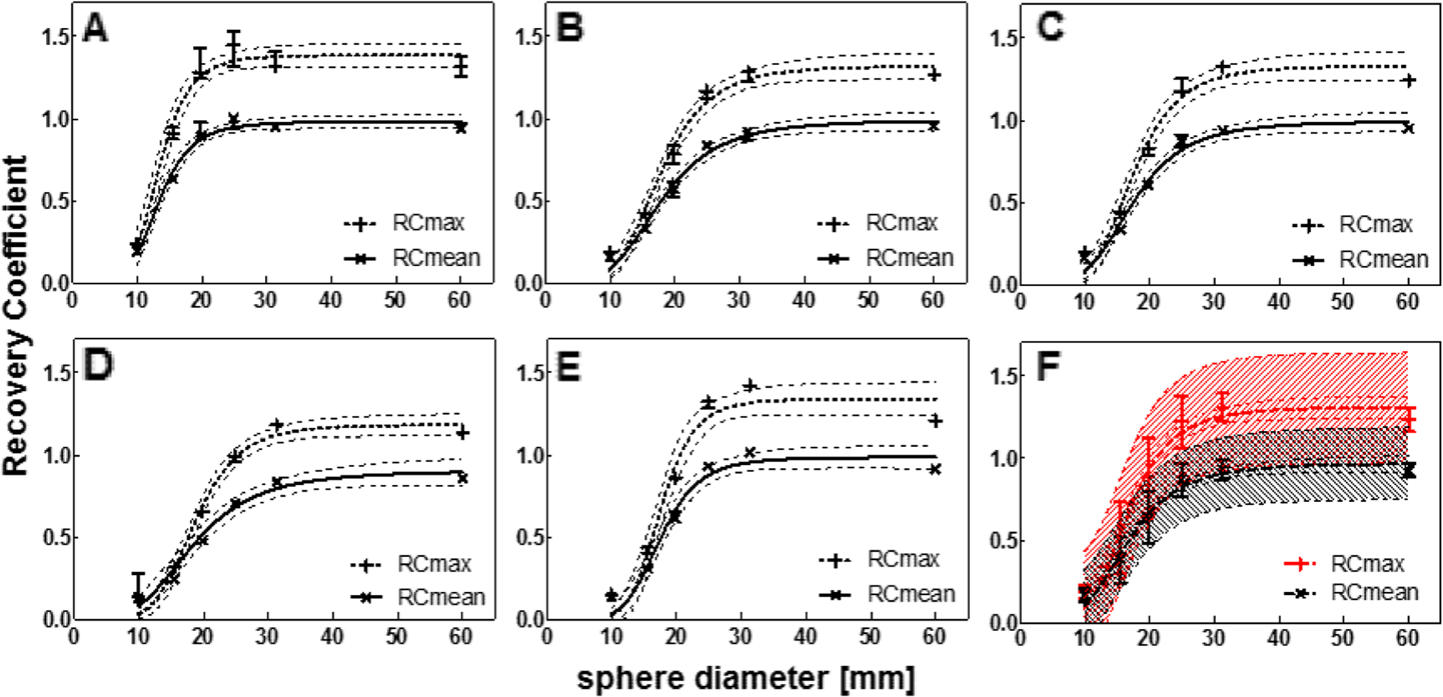
Recovery coefficient as a function of sphere diameter for all systems separately (**A**-**E**) and for all systems combined (**F**), for data reconstructed with a vendor specific algorithm. Median and range of three repetitive measurements per system. **A**) Discovery NM/CT 670 Pro; **B**) Symbia Intevo Bold with xSPECT Quant; **C**) Symbia Intevo Bold with Broad Quantification; **D**) Symbia T16 system 1; **E**) Symbia T16 system 2; **F**) Mean and standard deviation. All data were fitted with a 3-parameter logistic function (dashed line: 95% CI), for the combined data (F) also the 95% prediction interval is indicated (dashed area)

**Fig. 5:**
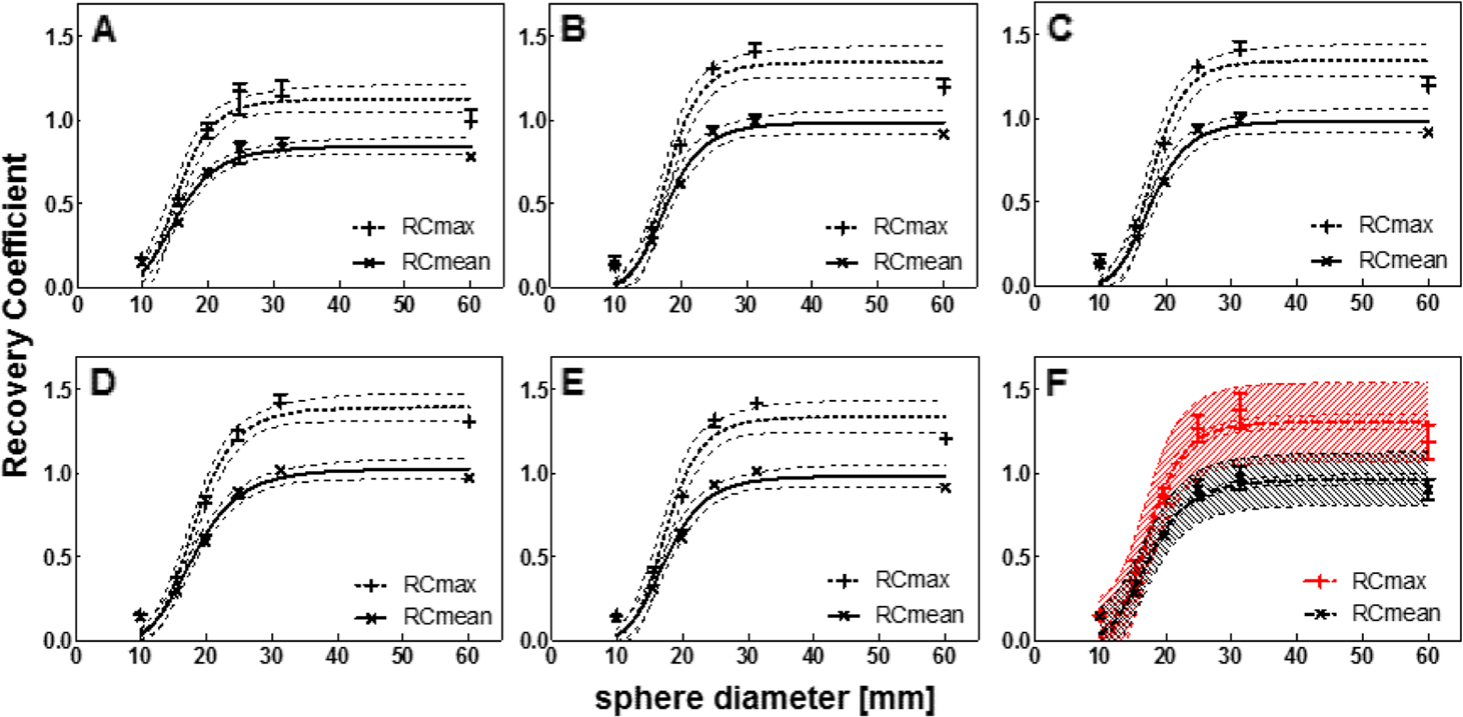
Recovery coefficient as a function of sphere diameter for all systems separately (**A**-**E**) and for all systems combined (**F**), for data reconstructed with a vendor neutral algorithm. Median and range of three repetitive measurements per system. **A**) Discovery NM/CT 670 Pro; **B**) Symbia Intevo Bold with xSPECT Quant; **C**) Symbia Intevo Bold with Broad Quantification; **D**) Symbia T16 system 1; **E**) Symbia T16 system 2; **F**) Mean and standard deviation for all systems combined. All data were fitted with a 3-parameter logistic function (dashed line: 95% CI), for the combined data (F) also the 95% prediction interval is indicated (dashed area

**Fig. 6:**
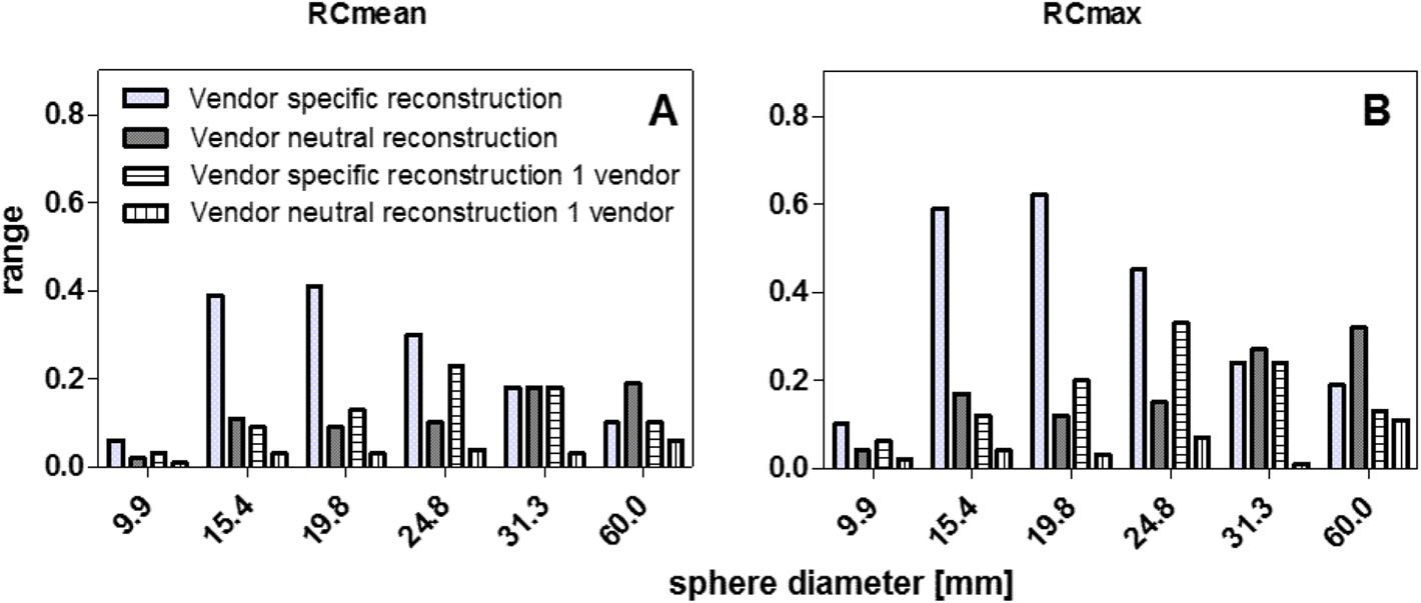
Comparison in range over all systems in RC_mean_ (**A**) and RC_max_(**B**) per sphere diameter for data reconstructed with a vendor specific algorithm versus a vendor neutral algorithm. Third and fourth columns give the same information but for systems of only one vendor, thus consisting of equal system hardware

The original article has been updated.
