# Supplementary material for: Variability in lutetium-177 SPECT quantification between different state-of-the-art SPECT/CT systems
Source: EJNMMI Phys. 2020 Feb 11;7:9. doi: 10.1186/s40658-020-0278-3 (PMC7013023; doi:10.1186/s40658-020-0278-3)
Supplement: Supplementary file 1 — Additional file 1:Table S1. Characteristics of ME collimators for all used SPECT/CT systems. Table S2. Acquisition settings of low dose CT protocols used for attenuation correction. Table S3. Cross-calibration protocols for dose calibrators to SPECT/CT system. [file 40658_2020_278_MOESM1_ESM.docx]

**Additional file 1**

Table S1: Characteristics of ME collimators for all used SPECT/CT systems

| System manufacturer | GE | Siemens |
| --- | --- | --- |
| Collimator name | Medium Energy General Purpose (MEGP) | Medium Energy Low Penetration (MELP) |
| Hole shape | Hexagonal | Hexagonal |
| Number of holes (x 1000) | 15.21 | 14.00 |
| Collimator hole diameter | 3.00 mm | 2.94 mm |
| Hole length | 58.00 mm | 40.64 mm |
| Septal thickness | 1.05 mm | 1.14 mm |
| Sensitivity for 67Ga @ 10 cm | 65 cps/MBq | 124 cps/MBq |
| Septal penetration | 2.0 % | 1.2 % |
| System resolution FWHM for 67Ga @ 10 cm for 3/5” crystal | 9.4 mm | 12.5 mm |

Table S2: Acquisition settings of low dose CT protocols used for attenuation correction

| System | Discovery NM/CT 670 Pro | Symbia Intevo Bold | Symbia T16 |
| --- | --- | --- | --- |
| Detector rows | 24 | 24 | 24 |
| Tube voltage [kVp] | 129 | 110 | 110 |
| Effective Tube current time product [mAs] | 25 | 30 | 23-30 |
| Reconstruction kernel | Standard | B08s | B08s medium sharp |
| Reconstructed slice thickness [mm] | 2.1 | 5.0 | 5.0 |
| Reconstruction FOV [mm] | 565 | 500 | 650 |
| Matrix size | 256x256 | 512x512 | 512x512 |

Table S3: Cross-calibration protocols for dose calibrators to SPECT/CT system

| System | System/Center specific cross-calibration method |
| --- | --- |
| Discovery NM/CT 670 Pro | Cylindrical phantom with diameter of 20 cm filled with ~80 kBq/ml ^177^Lu |
| Symbia Intevo Bold xSPECT Quant | NIST traceable calibration using a ^75^Se source (Calibrated Sensitivity Source (CSS)). |
| Symbia Intevo Bold Broad Quantification | Point source calibration with ^177^Lu using 5 - 30 kcps count rate, plus cylindrical phantom with diameter of 20 cm filled with ~80 kBq/ml ^177^Lu |
| Symbia T16 | Cylindrical phantom with diameter of 20 cm filled with ~80 kBq/ml ^177^Lu |
| All, Hermes SUV SPECT | Cylindrical phantom with diameter of 20 cm filled with ~80 kBq/ml ^177^Lu |
